# Comparative Analysis of rs12979860 SNP of the IFNL3 Gene in Children with Hepatitis C and Ethnic Matched Controls Using 1000 Genomes Project Data

**DOI:** 10.1371/journal.pone.0085899

**Published:** 2014-01-17

**Authors:** Giuseppe Indolfi, Giusi Mangone, Elisa Bartolini, Gabriella Nebbia, Pier Luigi Calvo, Maria Moriondo, Pier-Angelo Tovo, Maurizio de Martino, Chiara Azzari, Massimo Resti

**Affiliations:** 1 Paediatric and Liver Unit, Meyer Children's University Hospital of Florence, Florence, Italy; 2 Immunology Unit and Laboratory, Meyer Children's University Hospital of Florence, Florence, Italy; 3 U.O. Pediatria 2, Fondazione IRCCS Ca' Granda Ospedale Maggiore Policlinico, Milano, Italy; 4 Department of Pediatrics, Infectious Diseases Unit, University of Turin, Italy Regina Margherita Children's Hospital, Turin, Italy; 5 Department of Health Sciences, University of Florence, Florence, Italy; University of Sydney, Australia

## Abstract

The rs12979860 single nucleotide polymorphism located on chromosome 19q13.13 near the interferon L3 gene (formerly and commonly known as interleukin 28B gene) has been associated in adults with both spontaneous and treatment induced clearance of hepatitis C virus. Although the exact mechanism of these associations remains unclear, it suggests that variation in genes involved in the immune response against the virus favours viral clearance. Limited and preliminary data are available on this issue in children. The aim of the present study was to evaluate, in a representative cohort of children with perinatal infection, the potential association between rs12979860 single nucleotide polymorphism and the outcome of hepatitis C virus infection. Alleles and genotypes frequencies were evaluated in 30 children who spontaneously cleared the virus and in 147 children with persistent infection and were compared with a population sample of ethnically matched controls with unknown hepatitis C status obtained using the 1000 Genomes Project data. The C allele and the C/C genotype showed greater frequencies in the clearance group (76.7% and 56.7%, respectively) when compared with both children with viral persistence (C allele 56.5%, p = 0.004; C/C genotype 32.7%, p = 0.02) and with the ethnically matched individuals (C allele 59.7%, p = 0.02; C/C genotype 34.7%, p = 0.03). Children with the C/C genotype were 2 times more likely to clear hepatitis C virus relative to children with the C/T and T/T genotypes combined (odds ratio: 2.7; 90% confidence intervals: 1.3–5.8). The present study provides the evidence that the rs12979860 single nucleotide polymorphism influences the natural history of hepatitis C virus in children.

## Introduction

The rs12979860 single nucleotide polymorphism (SNP) on chromosome 19q13.13 near the *IFNL3* gene (formerly and commonly known as *IL28B*) has been associated in adults with both spontaneous and treatment induced clearance of hepatitis C virus (HCV) [1], [2]. Three interferon (IFN)-λ genes, *IFNL1*, *IFNL2* (formerly *IL29* and *IL28A*, respectively) and *IFNL3* reside downstream of the rs12979860 SNP [3]. IFN-λ genes encode the type III IFNs. Type III IFNs (IFN-λs) like type I IFNs (IFN-α and IFN-β) induce antiviral activity and suppress HCV replication *in vitro* and *in vivo* through activation of the JAK-STAT pathway which leads to induction of several IFN stimulated genes which are responsible for antiviral activity [3]. Although the exact molecular phenotype of the genetic association between rs12979860 SNP of the *IFNL3* gene and clearance of HCV remains unclear, it is evident that variation in genes involved in the immune response against the virus favours viral clearance [3].

There is evidence that the frequency of the C allele of the rs12979860 SNP varies markedly across ethnic groups [1]. The C allele is less frequent among individuals of African ancestry relative to those of European ancestry and East Asians [1]. It is well known that that the probability of being cured by the combined treatment with pegylated IFN-α and ribavirin increases accordingly [4]–[6]. It has been estimated that approximately half of the difference in sustained virological response rates to combined treatment with pegylated IFN-α and ribavirin among these different ethnic groups can be accounted for by the difference in the frequency of the C allele [2].

Most of the studies published on the association between rs12979860 SNP of the *IFNL3* gene and spontaneous clearance of HCV have been performed in adults. Only two studies on small cohorts of children are available so far on the topic [7], [8]. The natural history of hepatitis C in children is different when compared to adults [9], [10]. Liver disease severity tends to be milder in children than in adults. This is thought to be the consequence of the different interplay between the virus and the immune system of the child and it is particularly true when the infection is acquired perinatally from the mother in the context of the high level of immune tolerance of the developing immune system of the newborn [11].

To confirm the potential effect of rs12979860 SNP of the *IFNL3* gene on the outcome of HCV infection in terms of spontaneous clearance and chronic infection in children, this variant was genotyped in an HCV cohort comprised of mono-ethnic children who spontaneously cleared the virus or had persistent infection. Allele and genotype frequencies in this cohort were compared with a population sample of ethnically matched controls with unknown hepatitis C status obtained using the 1000 Genomes Project data [12].

## Materials and Methods

### Study design

This is a cross-sectional study. All the children, born to an HCV-infected mother and older than 30 months evaluated between January 2011 to December 2012 during routine follow-up visits for perinatal HCV infection were recruited. Only children whose parents or guardians provided written informed consent were enrolled and genotyped for the SNP rs 12979860. Retrospective data to define the source and the outcome of the infection were collected at the time of the enrolment. All the children enrolled were negative for hepatitis B surface antigen and for antibodies against human immunodeficiency virus. The ethnic origin of the children enrolled was self-reported by the parents or guardians. The study received the approval of the ethical committee of the Meyer Children's University Hospital of Florence.

### Definitions

Mother-to-child transmission of HCV was defined as transmission occurring during pregnancy or in the perinatal period from the HCV-infected mother to the foetus or to the child [13]. Maternal infection was confirmed basing on HCV antibodies testing in the last trimester before pregnancy or during pregnancy. Children were considered perinatally infected if HCV ribonucleic acid was detected in at least two serum samples at least three months apart during the first year of life, and/or when testing of antibodies against HCV was positive after 18 months of age [14]. Spontaneous clearance of HCV in children with perinatal infection is a well known phenomenon described in about 20% of the children with perinatal infection and occurring in the first 30 months of life [15]. Spontaneous clearance was therefore defined in children older than 30 months of age when polymerase chain reaction for HCV ribonucleic acid was negative and HCV antibodies were positive in at least three blood samples taken 6 months apart [9], [15], [16]. In the same age group positive polymerase chain reactions for HCV ribonucleic acid and HCV antibodies defined chronic hepatitis C [9], [15]. Only children older than 30 months of age were recruited. Children with spontaneous clearance of HCV were routinely followed up to 18 years of age as part of the local research protocol.

### Patients

One hundred and eighty six consecutive children were recruited. Nine of them (4.8%) were not enrolled as the parents did not provide the informed consent. Of the 177 children enrolled (95.2%), 30 consecutive children (16.9%) spontaneously cleared the virus and 133 (83.1%) were chronically infected.

### Comparison with individuals studied in the 1000 Genomes Project

Allele and genotype frequencies for the rs12979860 SNP were examined for concordance with the individuals studied in the 1000 Genomes Project (http://www.1000genomes.org), October 2010 release [12].

### Laboratory methods

Polymerase chain reaction for HCV was performed as previously described [15]. HCV antibodies were investigated by a third generation enzyme-linked immunosorbent assay (ELISA) (Ortho Diagnostic System Inc., Raritan, NJ) and confirmed by Western blot analysis (Innogenetics, Zwijndrecht, Belgium). *IFNL3* rs12979860 SNP genotyping was performed using Allelic Discrimination assays from Applied Biosystems following the instructions of the manufacturer. Genotyping was performed using Taqman custom-designed primers and probes as follows: forward primer GCCTGTCGTGTACTGAACCA, reverse primer GCGCGGAGTGCAATTCAAC, and probes TGGTTCGCGCCTTC (VIC) and CTGGTTCACGCCTTC (FAM) (Applied Biosystems). Before performing the PCR reactions DNA is added with Allelic Discrimination Assay Mix and TaqMan Universal PCR Master Mix to MicroAmp Optical 96-Well Reaction Plates (Applied Biosystems). Real-Time PCR reactions were performed using the 7500 Fast Real-Time PCR System. After preheating for 10 min. at 92°C, 40 cycles of 15 seconds at 95°C and one minute at 60°C followed. Data were analyzed using the ABI SDS software (Applied Biosystems).

### Statistical analysis

Data were processed with the SPSSX statistical package (SPSS, Inc., Chicago, IL). Two tailed P values were used and a p value <0.05 was defined as significant. Differences in frequencies were evaluated by chi^2^ test or Fisher's exact probabilities. Odds ratio (OR) and 90% confidence intervals (CI) were calculated. Age was presented as median and interquartile range (IQR). Hardy-Weinberg Equilibrium (HWE) was tested. A deviation from HWE was defined with a criterion of p value <0.05.

## Results

### HCV infected children

Thirty children (16.9%) spontaneously cleared the virus and 133 (83.1%) were chronically infected. The characteristics of the children enrolled are summarized in table 1.

**Table 1:** Characteristics of the children enrolled in the hepatitis C virus cohort.

	Spontaneous clearance	Chronic infection
	30 (16.9%)	147 (83.1%)
Male/female	17/13	79/68
Age years, median (IQR)	12.7 (6.8)	11 (6.9)
Genotype		
1	9	89
2	0	17
3	5	25
4	0	15
multiple genotypes	2	1
not determined	14	0
C allele	46 (76.7%)*	166 (56.5%)*
T allele	14 (23.3%)	128 (43.5%)
C/C genotype	17 (56.7%)**	48 (32.7%)
C/T genotype	12 (40%)	70 (47.6%)
T/T genotype	1 (3.3%)***	29 (19.7%)

Note: IQR, interquartile range; * p = 0.004; OR 2.5; 90%CI 1.4–4.6; ** C/C genotype versus C/T and T/T combined p = 0.02; OR = 2.7; 90%CI 1.3–5.8; *** C/C genotype versus T/T p = 0.01; OR = 10.3; 90%CI 1.3–217.8

### Data extracted from the 1000 Genomes Project database

**Figure 1:**
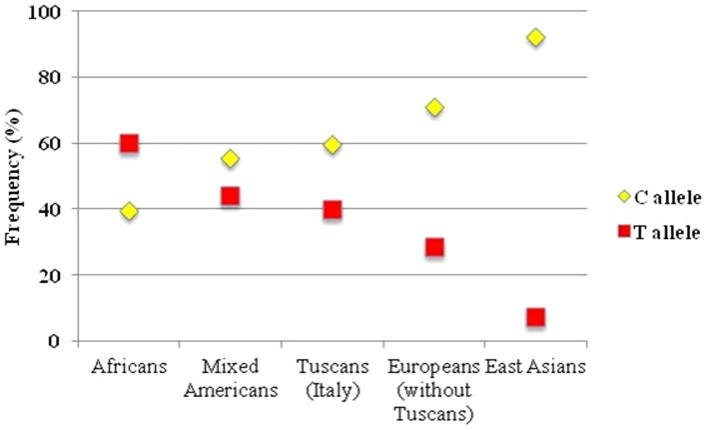
Frequency of rs12979860 C and T alleles in the diverse ethnic groups present in the 1000 Genomes Project database.

Alleles and genotypes frequencies for the rs12979860 SNP were available in the 1000 Genomes Project database for 1,092 individuals. Rs12979860 alleles and genotypes frequencies according to the ethnic origin are summarized in table 2. The frequencies of the C and T alleles in each population sampled is described in figure 1. The highest frequencies of the C allele and of the C/C genotype were found in East Asians (92.5% and 85%, respectively). Among the 379 individuals of European ancestry, 98 (25.9%) were Italians. Italy was the nation where the present study was carried out. The frequency of the C allele and of the C/C genotype were significantly lower in the Italians when compared with the remaining Europeans (for the C allele: 59.7% and 71.2%, respectively, p = 0.003; for the C/C genotype: 34.7% and 51.2%, respectively, p = 0.005).

**Table 2:** Rs12979860 alleles and genotypes frequencies in the 1,092 individuals enrolled in the 1000 Genomes Project database.

Population	Alleles n (%)		Genotypes n (%)			HWE	
	C	T	C/C	C/T	T/T		n
**EUROPEANS**	**517 (68.2)**	**241 (31.8)**	**178 (47)**	**161 (42.5)**	**40 (10.5)**	**0.68**	**379**
Iberians (Spain)	22 (78.6)	6 (21.4)	9 (64.3)	4 (28.6)	1 (7.1)	0.57	14
Utah residents (U.S.A) with Northern and Western European ancestry	127 (74.7)	43 (25.3)	49 (57.6)	29 (34.2)	7 (8.2)	0.37	85
Finnish	133 (71.5)	53 (28.5)	47 (50.5)	39 (42)	7 (7.5)	0.77	93
British	118 (66.3)	60 (33.7)	39 (43.8)	40 (45)	10 (11.2)	0.95	89
Tuscans (Italy)	117 (59.7)*	79 (40.3)	34 (34.7)**	49 (50)	15 (15.3)	0.69	98
Europeans (without Tuscans)	400 (71.2)*	162 (28.8)	144 (51.2)**	112 (39.9)	25 (8.9%)	0.63	281
**EAST ASIANS**	**529 (92.5)**	**43 (7.5)**	**243 (85)**	**43 (15)**	**–**	**0.41**	**286**
Japanese (Tokyo)	159 (89.3)	19 (10.7)	70 (78.7)	19 (21.3)	–	0.26	89
Chinese (Beijing)	179 (92.3)	15 (7.7)	82 (84.5)	15 (15.5)	–	0.41	97
Chinese	191 (95.5)	9 (4.5)	91 (91)	9 (9)	–	0.63	100
**AFRICANS**	**195 (39.6)**	**297 (60.4)**	**38 (15.4)**	**119 (48.4)**	**89 (36.2)**	**0.86**	**246**
Kenyans	95 (49)	99 (51)	19 (19.6)	57 (58.8)	21 (21.6)	0.08	97
Nigerians	59 (33.5)	117 (66.5)	11 (12.5)	37 (42)	40 (45.5)	0.59	88
Afro-Americans	41 (33.6)	81 (66.4)	8 (13.1)	25 (41)	28 (45.9)	0.52	61
**MIXED AMERICANS**	**202 (55.8)**	**160 (44.2)**	**55 (30.4)**	**92 (50.8)**	**34 (18.8)**	**0.68**	**181**
Mexicans	69 (52.3)	63 (47.7)	17 (25.8)	35 (53)	14 (21.2)	0.61	66
Puertoricans	71 (64.5)	39 (35.5)	22 (40)	27 (49.1)	6 (10.9)	0.59	55
Colombians	62 (51.7)	58 (48.3)	16 (26.7)	30 (50)	14 (23.3)	0.99	60

Note: HWE, Hardy-Weinberg Equilibrium. * p = 0.003. ** p = 0.005.

### Comparison of the allele frequencies

In the HCV cohort, the frequency of the C allele was 76.7% among children who spontaneously cleared the virus *versus* 56.5% among children with chronic infection (p = 0.004; OR: 2.5; 90%CI: 1.4–4.6). The frequency of the C allele among children in the HCV cohort who spontaneously cleared the infection was greater than that observed in the individuals of European ancestry but the difference did not reach the threshold of significance (75% *versus* 68.2%, p = 0.2). A significant difference was observed when the frequencies of C alleles of the children with spontaneous clearance and of Italians enrolled in the 1000 Genomes Project database were compared (76.7% *versus* 59.7%, p = 0.02; OR: 2.2; 90%CI: 1.2–4.1). The T-allele frequency was significantly greater in the chronically infected cohort compared with the European controls (43.5% *versus* 31.8%, p = 0.003).

### Comparison of genotype frequencies

Genotypes were in Hardy-Weinberg equilibrium in the cohort of children with HCV infection (p = 0.63), in individuals of European ancestry and Italians selected from the 1000 Genomes Project database (table 2). Children with the C/C genotype were 2 times more likely to clear HCV relative to children with the C/T and T/T genotype combined (OR: 2.7; 90%CI: 1.3–5.8, p = 0.02). No difference was observed for clearance of HCV between the C/T and T/T genotypes and between the C/C and the C/T. Clearance was more common in children with the C/C genotype than in those with T/T (OR: 10.3; 90%CI: 1.3–217.8, p = 0.01). The C/C genotype was found in 56.7% of the children with spontaneous clearance of HCV and 47% in individuals of European ancestry (p = 0.7). The C/C genotype was more common in children with spontaneous clearance of HCV than in the Italians present in the 1000 Genomes Project database (56.7% versus 34.7%, p = 0.03; OR 2.5; 90%CI 1.1–5.4).

## Discussion

The present study shows that the C allele and the C/C genotype of the rs12979860 SNP of the *IFNL3* gene are associated with spontaneous resolution of HCV infection in Italian children. The results of this study were obtained comparing the frequencies of the C and T allele and of the different genotypes of the rs12979860 SNP of the *IFNL3* gene between a cohort of children who spontaneously cleared or where chronically infected by HCV with ethnically matched controls. The rationale for this study was that, if the polymorphism influences natural clearance also in children, we would have obtained a frequency difference in these comparisons, since the children who naturally clear the virus will be excluded from the chronic infection cohort, thereby reducing the frequency of the allele that increases the likelihood of spontaneous clearance. The present results confirm the association demonstrated in adults [1]. Two preliminary paediatric studies were already published on the same topic [7], [8] that enrolled a limited number of patients (n = 15 and n = 26, respectively) and used the classical approach of comparing the frequencies of the C/C genotypes in children with clearance and chronic infection. In the present study a significantly higher number of children was enrolled and the value of the results was enforced and validated by the comparisons of alleles and genotypes frequencies among children with chronic infection, spontaneous clearance and, differently by previous studies, with a cohort of ethnically matched controls.

One of the risks of the interpretation of genome wide association studies is to find spurious associations [17]. For this reason, the data extracted by the 1000 Genomes Project in this study were analyzed for consistency with the previous data published on the topic [1]. A higher frequency of the C allele was confirmed in East-Asians, followed by the Europeans and Africans-Americans [1]. The patterns of genetic variation across different populations are shaped by history, environment, and stochastic processes. It is noteworthy that, among the Europeans, a group of individuals from Italy (the country were the study was carried out) had significantly lower frequencies of the C allele and of the C/C genotype than the other Europeans. The European individuals therefore were not considered representative of the cohort of HCV infected children and Italians were selected as reference. The rationale for this choice was the observation that different studies have shown that, despite low average levels of genetic differentiation among Europeans, there is a close correspondence between genetic and geographic distances [17], [18]. Two major axes of genetic variation have been observed within Europe, namely from North to South and from East to West and two representative populations have been identified, the CEU (Northwestern Europe) and the TSI populations (Tuscan Italians from Southern Europe). The pattern of variation of the rs12979860 SNP of the *IFNL3* gene observed among different European populations studied in the 1000 Genome Project is in line with the gradient of genetic variation described among Europeans following the North-Northwest/South-Southeast geographic axis [17]. When interpreting genome wide association studies the optimal solution is to select the geographically closer population as reference [17], [18]. Spurious associations can arise if genetic structure is not properly accounted for and the results of the present article are the proof of it.

The present study contributes to the understanding of the mechanisms of clearance of HCV infection in children with perinatal infection. Spontaneous clearance of HCV in children perinatally infected has been associated so far with a transaminase flare in the first year of life and with HCV genotype 3 infection [15], [19]. *IFNL3* is a human gene of the host system of innate antiviral defense. The outcome of HCV infection is dependent on the balance between innate immunity of the host and the multiple mechanisms to regulate and evade innate immunity evolved by HCV. Although early studies failed to find altered mRNA expression of *IFNL3* associated with different *IFNL3* genotypes, since the specificity of real-time PCR primers for *IFNL3* has increased (to differentiate between IFN-λ3 and the closely related IFN-λ2), it is now clear that SNPs of the *IFNL3* gene affect the expression of IFN-λ3 with the unfavourable allele resulting in less IFN-λ3 expression [20]. Furthermore, recently, a study showed that adults with the unfavourable *IFNL3* genotype have depressed innate immune function, particularly with respect to NK cells, suggesting that the decreased expression of *IFNL3* affects innate immunity and therefore clearance of HCV [21]. Quantitative and qualitative differences exist between adult and neonatal innate immune cells. Neonatal innate immune cells, for example, as compared with those of adults, have been described to produce lower levels of IFNs [22]. The activity of NK cells also differs between neonates and adults with many differences demonstrated in the pattern of cell-surface activating and inhibitory receptors [22]. Newborn and infant's immune responses are oriented towards immunological tolerance and neonatal innate immune defences are skewed towards protection against extracellular bacterial pathogens rather than intracellular bacterial pathogens and viruses [11]. Despite innate immunity in neonates and infants can be condidered physiologically depressed, it was of interest to evaluate if it still plays a role, in children as in adults, in spontaneous elimination of the virus. The results of the present study, confirming that the rs12979860 SNPs influences the natural history of hepatitis C virus in children, support that hypothesis.

SNPs of the *IFNL3* gene have been associated in adults with both spontaneous clearance of HCV infection and response to treatment with combined dual therapy with pegylated IFN and ribavirin. These results suggest that IFN-λ3 has a key role in determining both treatment-induced and spontaneous clearance of HCV. The association between rs12979860 SNP of the *IFNL3* gene and spontaneous clearance in children and the homology of this result with those obtained in adults, supports the hypothesis that *IFNL3* gene polymorphisms should be evaluated extensively as pretreatment predictor of virological response also in children.

In conclusion, the present result demonstrates that the frequencies of the C allele and the C/C genotype of the rs12979860 SNP of the *IFNL3* gene are higher in a cohort of children who spontaneously cleared HCV infection when compared to children with chronic hepatitis C and ethnically matched controls. The rs12979860 SNP of the *IFNL3* gene influences spontaneous resolution of HCV infection in Italian children.
